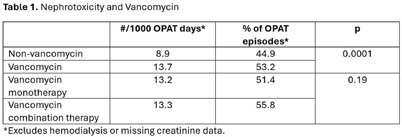# Systematic Electronic Capture of Outpatient Parenteral Antimicrobial Therapy (OPAT) Adverse Events, Implications for Performance

**DOI:** 10.1017/ash.2025.396

**Published:** 2025-09-24

**Authors:** Colin Samoriski, William DePasquale, Alexandra (Sasha) Yamshchikov

**Affiliations:** 1 University of Rochester Medical Center

## Abstract

**Background:** OPAT has emerged as an effective modality for continued treatment of serious infections outside the hospital, requiring complex care coordination and close monitoring for patient safety. Despite increasing availability of OPAT services nationwide, monitoring and benchmarking of treatment-related adverse events, patient outcomes, and program quality remain labor intensive and inconsistent across programs. **Method:** A previously reported OPAT-specific bundle of modifications to an Epic® Systems Corporation electronic health record (EHR) at a large academic OPAT program was leveraged to develop a model for longitudinal electronic monitoring and reporting of OPAT adverse event and safety outcomes data. An EHR-based SQL report evaluated mortality within 1 year of OPAT start, as well as intravascular access device (IVAD) occlusions (defined as documented intracatheter administration of alteplase), IVAD associated deep venous thromboses (DVT) (defined by 212 Upper Extremity DVT ICD-10 codes via custom SNOMED CT concept hierarchy grouper), anaphylaxis (defined by ICD-10 codes T78.2 and T88.6), and nephrotoxicity (defined as >0.3 increase or >1.5 times increase in baseline serum creatinine) while on OPAT. Hospital readmissions, emergency department utilization, non-anaphylactic allergic reaction (defined as documentation of new allergy to OPAT antibiotic), were evaluated while on OPAT or within 30 days of conclusion. **Result:** Total of 5190 OPAT episodes (10/18/2018 to 12/3/2024) in 4213 unique patients were examined (Figure 1). Bone/joint infection and bacteremia were most frequent indications for OPAT (Figure 2), with vancomycin, ceftriaxone, and cefazolin most common antibiotics (Figure 3). Rates of adverse events over time (Figure 4) were notable for high prevalence of nephrotoxicity affecting 2075 (40%) of all episodes, and demonstrating significant association with vancomycin therapy, although no difference was observed between vancomycin monotherapy and vancomycin-containing combination regimens (Table 1). The highest incidence of non-anaphylactic allergic reactions was noted with nafcillin, affecting 8.51% courses (rate 2.51/1000 Nafcillin OPAT days, p=0.018) and cefepime, affecting 4.18% courses, (rate 1.36/1000 Cefepime OPAT days, p=0.008). One-year mortality following enrollment into OPAT was 11%. **Conclusions:** Leveraging a robust informatics and reporting infrastructure may allow for consistent and ongoing capture of OPAT-related adverse events and outcomes. More studies are needed to validate standardized approaches for longitudinal evaluation of OPAT program safety and quality, supported by development of regional and national performance benchmarks.

**Figure:**
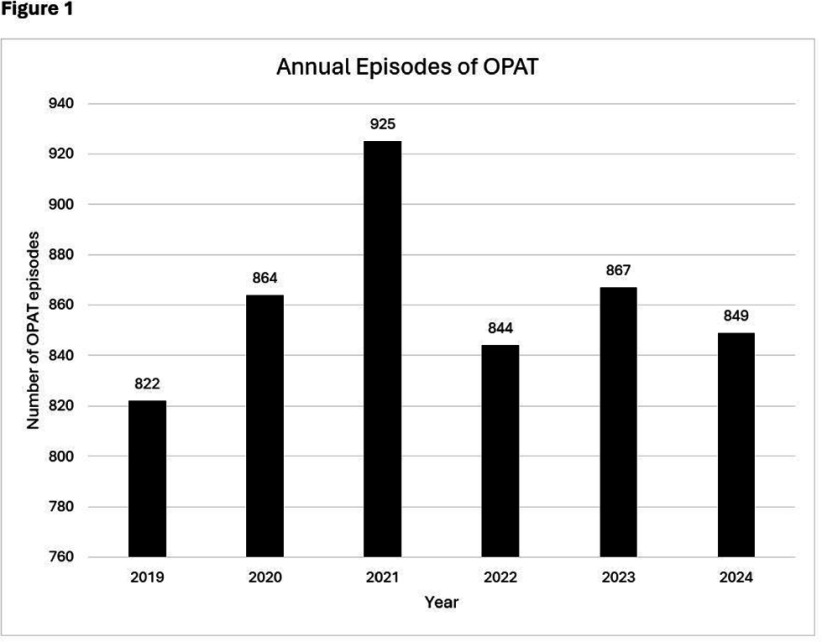

**Figure:**
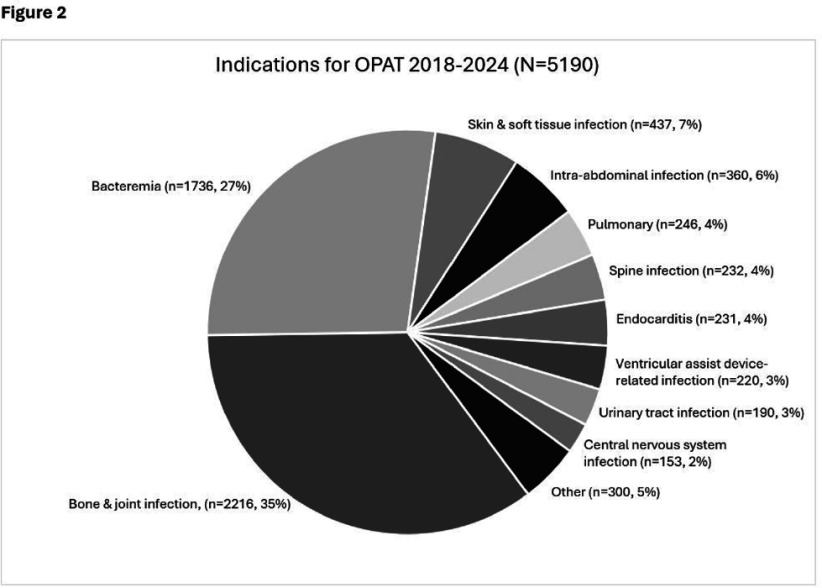

**Figure:**
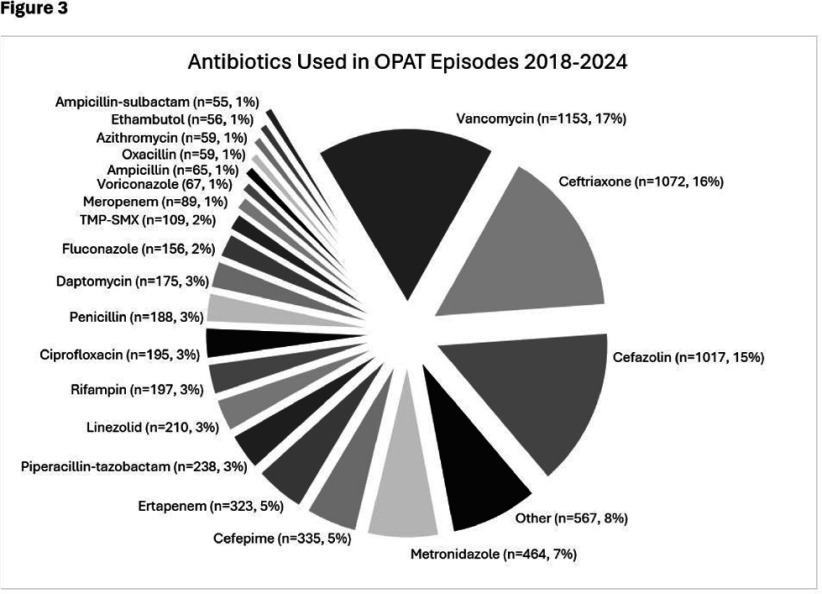

**Figure:**
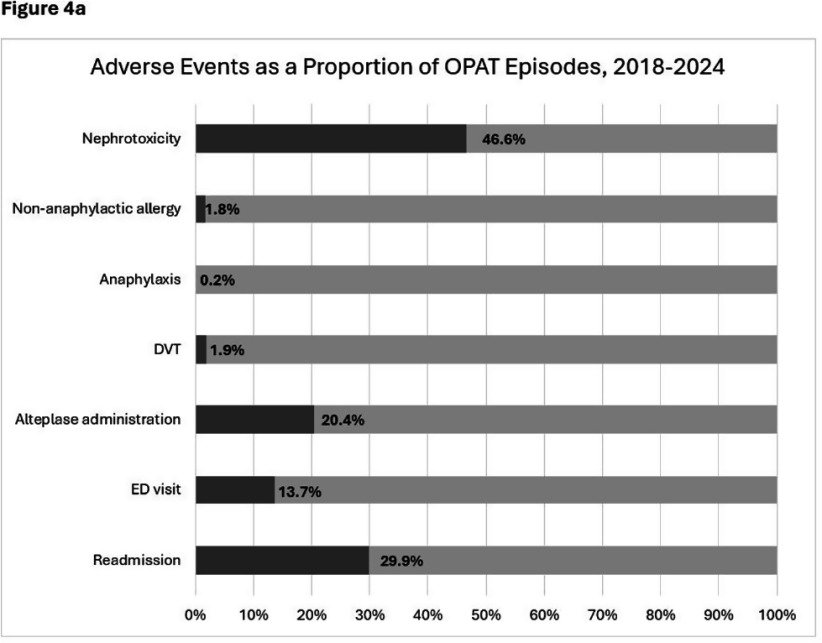

**Figure:**
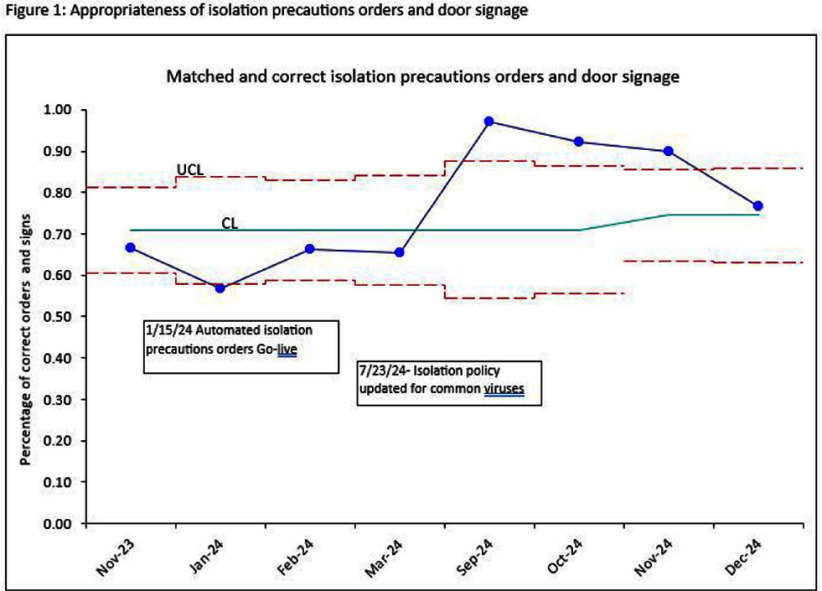

**Figure:**